# Human Pluripotent Stem Cell Colony Migration Is Related to Culture Environment and Morphological Phenotype

**DOI:** 10.3390/life14111402

**Published:** 2024-10-31

**Authors:** Vitaly V. Gursky, Alina S. Chabina, Olga A. Krasnova, Anastasiia A. Kovaleva, Daria V. Kriger, Michail S. Zadorsky, Konstantin N. Kozlov, Irina E. Neganova

**Affiliations:** 1 Institute of Cytology, 194064 Saint Petersburg, Russia; 2 Ioffe Institute, 194021 Saint Petersburg, Russia; 3 Mathematical Biology and Bioinformatics Lab, Peter the Great Saint Petersburg Polytechnic University, 195251 Saint Petersburg, Russia

**Keywords:** human pluripotent stem cells, migration, morphological phenotype, diffusion models

## Abstract

Human pluripotent stem cells (hPSCs) are an important tool in the field of regenerative medicine due to their ability to differentiate towards all tissues of the adult organism. An important task in the study of hPSCs is to understand the factors that influence the maintenance of pluripotent and clonal characteristics of colonies represented by their morphological phenotype. Such factors include the ability of colonies to migrate during growth. In this work, we measured and analyzed the migration trajectories of hPSC colonies obtained from bright-field images of three cell lines, including induced hPSC lines AD3 and HPCASRi002-A (CaSR) and human embryonic stem cell line H9. To represent the pluripotent status, the colonies were visually phenotyped into two classes having a “good” or “bad” morphological phenotype. As for the migration characteristics, we calculated the colony speed and distance traveled (mobility measures), meandering index (motion persistence measures), outreach ratio (trajectory tortuosity characteristic), as well as the velocity autocorrelation function. The analysis revealed that the discrimination of phenotypes by the migration characteristics depended on both the cell line and growth environment. In particular, when the mTESR1/Matrigel culture environment was used, “good” AD3 colonies demonstrated a higher average migration speed than the “bad” ones. The reverse relationship between average speeds of “good” and “bad” colonies was found for the H9 line. The CaSR cell line did not show significant differences in the migration speed between the “good” and “bad” phenotypes. We investigated the type of motion exhibited by the colonies by applying two diffusion models to the mean squared displacement dynamics, one model corresponding to normal and the other to anomalous diffusion. The type of diffusion and diffusion parameter values resulting from the model fitting to data demonstrated a similar cell line, environment, and phenotype dependency. Colonies mainly showed a superdiffusive behavior for the mTESR1/Matrigel culture conditions, characterized by longer migration steps compared to the normal random walk. The specific properties of migration features and the patterns of their variation demonstrated in our work can be useful for the development and/or improvement of automated solutions for quality control of hPSCs.

## 1. Introduction

There is currently a growing collection of patient-specific induced pluripotent stem cell lines (hiPSCs) around the world. These cells are unique in their characteristics due to their pluripotency, that is, the ability to differentiate into all tissues of the adult body. Moreover, these cells were found to be useful in disease modeling, pharmacological drug testing, modeling development processes, and many others [1,2,3]. In the quest to choose the best cellular model based on hiPSCs, it is important to choose the best hiPSC clone since it is well known that different hiPSC lines vary in their morphological and proliferative characteristics when cultured in different laboratories. Moreover, not all generated hiPSC clones are equally capable of further directed differentiation, which complicates their transition to clinical trials. In addition, clinical applications most likely would require a large number of hiPSCs or their progenies, meaning the need for stricter automated control of the cell cultures. Thus, the development of mathematical methods for analyzing cell cultures using automatic approaches based on machine and deep learning models is currently of great importance for the successful and safe further translation of hiPSCs to clinics.

Previously, we employed the bright-field images to define a set of morphological parameters of a hPSC colony that correlate well with its morphological phenotype, defined as “bad” or “good” [4]. Our previous analysis demonstrated a direct link between morphological “portrait” and pluripotency level of hPSCs. This analysis was based on the hPSC morphological phenotypes as judged by the expression of eleven pluripotency markers, differentiation properties towards the three germ layers, and proteomic analysis [4]. As we have assessed these phenotypes on the same cell lines in the previous experiments, in the present study, we chose not to use any invasive methods when tracking colony migration. Earlier, by applying machine learning methods, we developed a model for image prediction and recognition that classified the colony phenotype as “good” or “bad” with 99% accuracy, reflecting its pluripotency [5,6]. These works were in line with many other studies utilizing modeling and artificial intelligence to decipher hPSC phenotypic characteristics (see, for example, a recent review [7]). Accordingly, hPSC colonies in the present study were divided into those with “bad” or “good” morphological phenotypes. Moreover, our proteomic analysis of the two groups showed that cytoskeletal elements demonstrated the most differences in their proteomic landscapes across the groups as compared to other proteins [7]. This gave us a reason to assume that hPSCs with different morphological phenotypes could have different migration characteristics and behavior.

We set out to characterize the migration of the two types of colonies with “bad” and “good” phenotypes. The migratory behavior of cells can be analyzed by multiple metrics, which bear physiological significance [8,9]. For that purpose, we calculated the colony speed and distance traveled (mobility measures), meandering index (motion persistence measures), outreach ratio (trajectory tortuosity characteristic), as well as the velocity autocorrelation function for two hiPSCs lines (AD3 and CaSR) and commonly used human embryonic stem cell line, H9. Our main aim was to quantify a possible difference between colonies of distinct phenotypes using these migration characteristics.

Using a set of migration parameters is a reliable approach for understanding the migration patterns demonstrated by moving cells since different parameters can be informative for different conditions [10,11,12,13]. In addition to such parametric analysis, various anomalous phenomena of cell movement were previously described using modeling [14,15]. As cellular movement involves the coordinated action of several components, it cannot be fully characterized by a model of the normal Brownian motion and requires models of anomalous diffusion, in which migration involves various characteristic phases with spatiotemporal correlations [15]. We followed this approach and investigated the type of motion exhibited by hPSC colonies of different phenotypes in the framework of known diffusion models.

Despite significant progress achieved recently in the creation of Xeno-free media for the cultivation of the hiPSCs, there is still a lack of robust methods for their clonal expansion. To date, mechanisms regulating the heterogeneous phenotype of hiPSCs colonies and potential relationships to the functional properties of the clones with different phenotypes are poorly studied. Moreover, how this phenomenon is dependent on cultural conditions attracts less attention. In this work, we analyzed colony migration under two different culture conditions to characterize possible condition-dependent variability. We show that the hPSC phenotypes could be discriminated across all studied cell lines in terms of various migration parameters. Further integration of these results into a previously created model based on morphological image processing [4] could improve the automated and non-invasive hPSC culture assessment.

## 2. Materials and Methods

### 2.1. Cell Culture and Propagation

Human embryonic stem cell H9 cell line (WiCell, Madison, WI, USA), hiPSC line AD3 [16], and patient-specific hiPSC line HPCASRi002-A (CaSR) [17] were passaged on 6-well plates coated with hESC-qualified Matrigel Matrix (Corning Matrigel Matrix, Life Sciences) with expansion at a 1:4 split ratio using 0.02% EDTA (Versene) dissociation solution and 10 µM ROCK inhibitor (Y-27632; StemCell Technologies, Vancouver, BC, Canada). 2 mL of mTERSR1 media (StemCell Technologies) or Essential E8 (Thermo Fisher Scientific, Waltham, MA, USA) with corresponding matrix Geltrex/GT (Thermo Fisher Scientific) were used per well daily for all cultures. All cell cultures were checked daily. All cultures were kept at standard condition for 5 days at 37 °C with 5% CO_2_ atmosphere and 21% O_2_ according to WiCell Inc. protocols. All used cell lines were at their middle passages, i.e., p 26–30.

For time-lapse recording, image capture was performed with the CellVoyager CQ1 High-Content Analysis System (Yokogawa, Japan) at every 15 min time frames, using a 10× dry objective lens with a numerical aperture of 0.4. The images had a pixel size of 2528 × 2136 and a physical pixel size of 0.6667 μm along the x and y axes.

### 2.2. Calculation of Colony Trajectories and Migration Measures

Colony migration was tracked in the stack of images with growing colonies at different times (15 min time window) using tools implemented in ImageJ (Fiji). Image registration was first applied using the Linear Stack Alignment with SIFT plugin, removing unnecessary shifts of the focus area which might happen during imaging. After registration, trajectories of colony migration were obtained from the image stack by applying the Manual Tracking plugin to each colony. At each time, the center of the tracked colony was manually chosen, and the trajectory joining the 2D coordinates of the center points through time was saved as a csv file. The tracking of a colony was stopped if it merged with another colony.

The colony migration measures reported in the text were calculated for each trajectory using Wolfram Mathematica software Version 12.0.0 [18]. An instant colony speed was the distance between subsequent two points (instant distance) in the trajectory divided by the time (15 min) between these points. The mean colony speed was calculated by averaging the instant speed values along the colony trajectory. The total distance *d*_tot_(*t*) traveled by a colony by time *t* was the sum of all instant distances along the trajectory up to that time. The meandering index of trajectory by time *t* was calculated as a ratio of distance *d*_net_(*t*) between the initial point and point at time *t* to the total distance *d*_tot_(*t*) traveled by that time. The outreach ratio of trajectory by time *t* was calculated as a ratio of maximal distance *d*_max_(*t*) between the initial point and all other points by time *t* to the total distance *d*_tot_(*t*) traveled by that time.

The velocity autocorrelation function ρ(*t*) was calculated as follows:

$$\rho \left(t\right)=\langle v_{x}(t+t_{0})v_{x}(t_{0})\rangle +\langle v_{y}(t+t_{0})v_{y}(t_{0})\rangle ,$$

where *v_x_* and *v_y_* are the *x*– and *y*–coordinate projections, respectively, of the colony velocity vector, *t*_0_ is a fixed starting time, and $\langle \ldots \rangle$ denotes a combined average over all starting times *t*_0_ and all trajectories. The exponential approximations for the velocity autocorrelation function, expected from the normal diffusion assumptions, were calculated by fitting the function a*exp(–b*t) with arbitrary *a* and *b* to the data values of ρ(*t*).

### 2.3. Diffusion Models and MSD Fitting

We considered the following fractional Klein–Kramers equation to describe the dynamics of probability distribution *P*(*x*, *v*, *t*) for the colony to be in position *x* and time *t* and have velocity *v* [15,19]:

$$\frac{\partial P(x,v,t)}{\partial t}=-\frac{\partial }{\partial x}\left(vP\right)+\frac{\partial^{1-\alpha }}{{\partial t}^{1-\alpha }}\gamma_{\alpha }\left(\frac{\partial }{\partial v}\left(vP\right)+v_{th}^{2}\frac{\partial^{2}}{{\partial v}^{2}}P\right),$$

where α determines the order of the fractional derivative in the equation, $\gamma_{\alpha }$ is the parameter associated with damping, and $v_{th}^{2}=k_{B}T/M$ is the mean thermal velocity, where $k_{B}$ is the Boltzmann constant, *T* is the temperature, and *M* is the mass of the diffusive object. The mean squared displacement (MSD) in the diffusion model based on the FKK equation takes the following form in the presence of uncorrelated noise of variance $\sigma^{2}$ generated by possible measurement errors and biological activity [15]:

(1) $$MSD\left(t\right)=4v_{th}^{2}t^{2}E_{\alpha ,3}\left(-\gamma_{\alpha }t^{\alpha }\right)+{\left(2\sigma \right)}^{2},$$

where $E_{\alpha ,3}$ is the generalized Mittag–Leffler function $E_{\alpha ,\beta }$ with β=3. In the limit t→∞, $MSD\left(t\right)\sim D_{\alpha }t^{2-\alpha }$ with the diffusion coefficient $D_{\alpha }=v_{th}^{2}/\gamma_{\alpha }$.

A special case of the FKK model for α=1 is the Ornstein–Uhlenbeck process, which represents a model of the Brownian motion for an object with mass and friction [20]. The MSD in this case takes the following form:

(2) $$MSD\left(t\right)=\frac{4v_{th}^{2}}{\gamma_{1}^{2}}\left(\gamma_{1}t+e^{-\gamma_{1}t}-1\right)+{\left(2\sigma \right)}^{2}.$$

The fitting of functions (1) and (2) to the MSD values from data was performed by numerical minimization of the cost function representing the weighted residual sum of squares between function values and data, with the MSD variance at each time point used as weights. Values of parameters α, $\gamma_{\alpha }$, $v_{th}$, and σ were optimized during the numerical minimization involving Equation (1), and $\gamma_{1}$, $v_{th}$, and σ in the case of Equation (2). 20 independent minimization runs were performed for each condition (cell line, culture condition, and morphological phenotype) using the NMinimize function in Wolfram Mathematica software [18].

We compared the MSD fitting quality between the two diffusion models using the following Akaike information criterion (AIC), which accounts for both the values of minimized cost function and the number of free parameters *k*: AIC=2k−2log⁡L, where *L* is the maximum value of the likelihood function, and (–2log*L*) in this formula equals the minimal value of the cost function considered in our study [21]. A smaller AIC value corresponds to a better model.

### 2.4. Statistical Analysis

Comparison of means in the statistical analyses was performed by applying the Mann–Whitney test. The Kolmogorov–Smirnov test was used to compare the probability distributions. All statistical analyses were performed using Wolfram Mathematica software [18].

## 3. Results

### 3.1. General Plan of the Study

**Figure 1:**
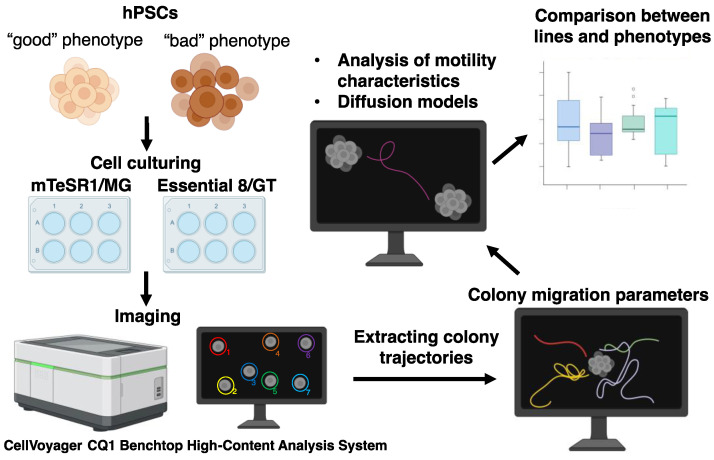
Study design. More details are given in the text.

The general scheme of this work is presented in Figure 1. hPSCs of three cell lines were cultured under two conditions, which consisted of two media/matrix combinations (mTESR1/MG and E8/GT, see Section 2). We considered the following cell lines: the human induced pluripotent stem cell (hiPSC) line AD3, patient-specific hiPSC line CaSR, and human embryonic stem cell line H9. All cell colonies were phenotyped as either “good” or “bad,” according to the visually assessed morphological properties representing the colony pluripotency status; the criteria for defining phenotype on the same cell lines were described in detail in [4]. “Good” colonies contained non-differentiated cells, while cells in “bad” colonies showed signs of starting or ongoing differentiation. The differentiation processes in the “bad” colonies manifested themselves through multiple changes in the morphology of cells and colonies (examples of “good” and “bad” colonies are given in Figure S1).

**Figure 2:**
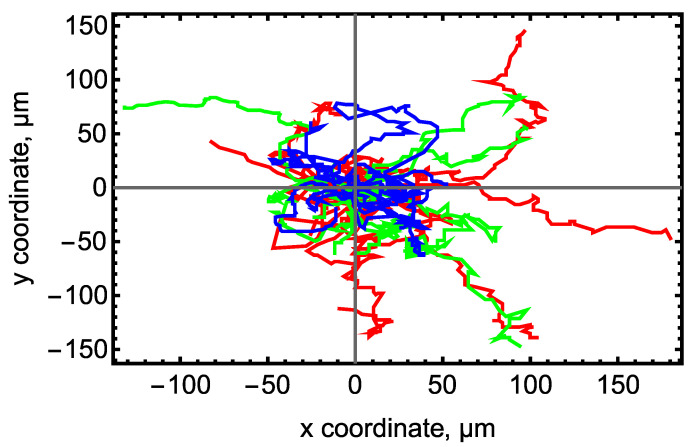
Examples of colony trajectories extracted from bright-field images of AD3 (red), CaSR (green), and H9 (blue) cell lines cultivated under mTESR1/MG conditions. Each line in the figure connects the coordinates (*x*, *y*) of the center of one colony during its migration. Ten randomly selected trajectories per line are shown. All trajectories are shifted to have the initial coordinates (*x*, *y*) = (0, 0).

In the next step, we performed time-lapse imaging of growing colonies using bright-field microscopy. We extracted 2D trajectories of colony migration from the stack of bright-field images acquired at multiple time points with the 15 min interval. For this purpose, the coordinates of the center of each colony were located in all images in the stack. Each trajectory thus represented a set of coordinates (*x*, *y*) of the colony center collected with the 15 min interval (Figure 2; Video S1). Table 1 shows the total number of colony trajectories under analysis for each cell line, growth condition, and phenotype. Colonies could merge with other colonies during migration, so each colony was tracked only until a possible merging event. As a consequence, the collected trajectories had different lengths, and the number of colonies under analysis reduced over time (Figure S2).

We used the trajectories to calculate several colony migration parameters that characterized the colony mobility, the persistence in its motion, the compactness of trajectories, and the correlation between steps in the motion (Table 2). As the mobility characteristics, we calculated the colony speed and the total distance *d*_tot_ traveled by the colony at a given time. The meandering index represented the tortuosity of trajectories or persistence of colony motion. We used the outreach ratio as a parameter characterizing the compactness of trajectories. As a measure of correlation between different steps in motion, we calculated the velocity autocorrelation function. Finally, we used the mean squared displacement (MSD) as an additional mobility measure.

In the next step, we performed two separate analyses based on these migration parameters. In Section 3.2, we compared the dynamics of the migration parameters for different lines, culture conditions, and colony phenotypes. Our main goal was to check whether the “good” and “bad” colonies could clearly be distinguished by the values of their migration parameters. If such a difference existed, we sought to determine whether it was qualitatively similar across cell lines and culture conditions. In Section 3.3, we used the MSD from Table 2 to infer the diffusion characteristics demonstrated by the hPSC colonies and compared these characteristics across different conditions.

### 3.2. Variability of Migration Characteristics Across Morphological Phenotypes

#### 3.2.1. Mean Colony Speed

We investigated how well the migration measures discriminate between the two morphological phenotypes and how this discrimination preserves across cell lines and culture conditions. The distribution of the instantaneous speed values exhibits long tails for both “good” and “bad” colonies and remains qualitatively similar across all cell lines and culture conditions (Figure S3). This indicates that most motion events occur with a relatively small speed, with the median speed in the range of 0.09–0.22 µm/min. Motion events with up to an order of magnitude larger speed happen with smaller frequencies. Despite the qualitative similarity, the distributions are quantitatively different for different phenotypes (*p* < 0.0001; Figure S3), implying possible different mobility parameters for “good” and “bad” colonies.

**Figure 3:**
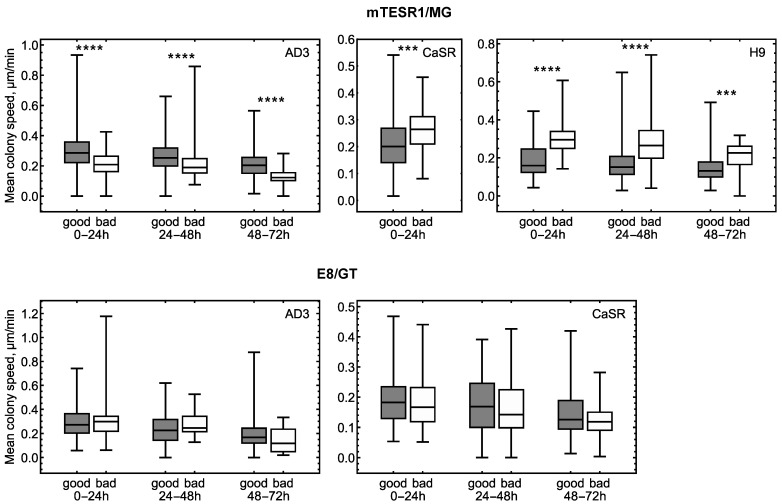
Box plots for the mean colony migration speed within three time intervals for all cell lines, culture conditions, and phenotypes. The averaging within a time interval was performed over all trajectories that either ended within that interval or lasted longer. Statistically significant difference between phenotypes is marked by stars: *p* < 0.001 (***) and *p* < 0.0001 (****). The absence of stars in the panels indicates statistically nonsignificant difference.

Unfolding the analysis of mobility at the individual colony level, we averaged the speed that colonies exhibited within three time intervals (0–24 h, 24–48 h, and 48–72 h) and considered how this speed differed between phenotypes. For the mTESR1/MG culture conditions, “good” AD3 colonies have a larger average speed than the “bad” colonies, while the opposite is true for CaSR and H9 colonies (Figure 3). A similar analysis for the E8/GT growth conditions showed no significant difference in the average speed between phenotypes (Figure 3).

The trajectories of different colonies generally had different lengths since the colonies were only tracked until possible merging events, and these events could occur at different times or not at all. The time averaging for each time interval in Figure 3 was performed for trajectories that either ended within the selected time interval or lasted longer. We conducted a similar analysis of mean speeds but excluded trajectories that ended before the end of the given time interval. This analysis yielded the same conclusions about the differences between the phenotypes (Figure S4). The only deviation in that case was the loss of statistically significant difference between phenotypes for the CaSR colonies under mTESR1/MG (Figure S4). For this line and this culture condition, “bad” colonies are, on average, faster than “good” ones only at early times. However, the small size of some samples in the analysis (Figure S4) makes the conclusions less robust.

We verified that “good” and “bad” colonies had different average speeds even after controlling for the variation in trajectory length. For this purpose, we selected only those colonies of both phenotypes that had trajectories of exactly the same length and compared their mean speeds (Figure S5). The observed difference between phenotypes was preserved in this analysis, and no trajectory length preference was found in this difference.

**Figure 4:**
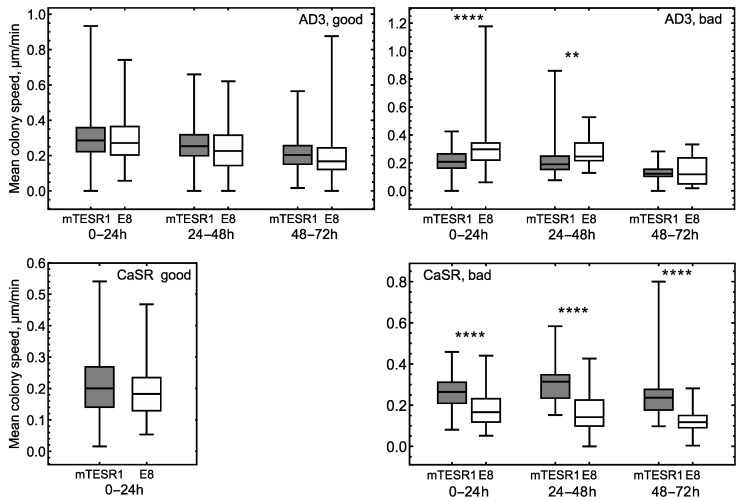
Box plots for the mean colony migration speed within various time intervals for “good” (**left panels**) and “bad” (**right panels**) colonies under two culture conditions. Top panels present results for AD3, and the bottom panels for CaSR. The averaging within a time interval was performed over all trajectories that either ended within that interval or lasted longer. Statistically significant difference between the mTESR1/MG and E8/GT conditions is marked by stars: *p* < 0.01 (**) and *p* < 0.0001 (****). The absence of stars in the panels indicates statistically nonsignificant difference.

We combined the box plots for AD3 colonies from Figure 3 in a way suitable for comparison of colony mobility in different culture conditions (Figure 4). The resulting figure showed that the mean speed of “good” colonies was, on average, the same for the two culture conditions. On the other hand, “bad” AD3 colonies under E8/GT showed higher motility compared to mTESR1/MG at early and middle times, and the difference vanished at later times. The mean speeds were also different between the culture conditions for “bad” CasR colonies, but in this case, the mTESR1/MG conditions provided more mobile colonies (Figure 4). This result illustrates that the mobility characteristics of “good” colonies are less variable with changing culture conditions compared to “bad” colonies.

#### 3.2.2. Total Distance

**Figure 5:**
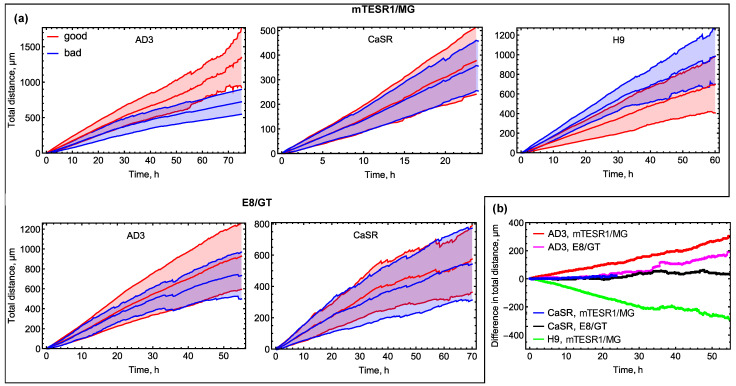
Total distance *d*_tot_(*t*) covered by colonies up to time *t* during migration. (**a**) Total distance as a function of time for all cell lines, culture conditions, and phenotypes. The shaded area depicts the mean ± standard deviation range, and the center line within the shaded area represents the dynamics of the mean value. Table S1 contains the total numbers of colonies used for testing a difference in the mean value of *d*_tot_(*t*) between the groups of “good” and “bad” colonies; (**b**) Dynamics of the difference between mean *d*_tot_(*t*) for “good” colonies and mean *d*_tot_(*t*) for “bad” colonies.

These mobility properties of “good” and “bad” colonies are also reflected in the total distance *d*_tot_ covered by colonies during migration (Figure 5). Faster-moving “good” AD3 colonies migrate, on average, on longer distances than “bad” ones under mTESR1/MG, and the opposite holds true for H9 colonies (Figure 5a). The separation of colonies with different morphological phenotypes in these cell lines was clear already at 24 h (*p* < 0.0001) and remained later on. There was no significant difference in the mean total distance between “good” and “bad” CaSR colonies at any time under mTESR1/MG (*p* > 0.05). This implies that the observed slightly higher average speed of “bad” colonies in this line at early times does not lead to essentially longer distances covered by these colonies. For the AD3 and CaSR lines under E8/GT, we could not statistically distinguish between phenotypes considering the total distance as a marker. This is in accordance with what we found for the average colony speed. Despite this, we observed a small gradual deviation of the average total distance covered by AD3 “good” colonies from “bad” colonies towards higher values (Figure 5a). The dynamics of the difference between average values of *d*_tot_ for “good” and “bad” colonies illustrates the higher mobility of “good” colonies compared to “bad” ones for AD3 (Figure 5b). For H9, this dynamic shows the higher mobility of “bad” colonies (Figure 5b). For CaSR, the two phenotypes have rather equal mobility (Figure 5b).

#### 3.2.3. Meandering Index and Outreach Ratio

**Figure 6:**
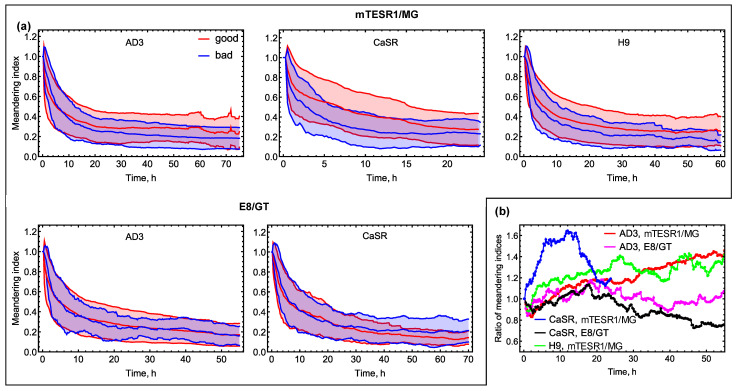
Dynamics of meandering index (*d*_net_(*t*)/*d*_tot_(*t*)) during migration. (**a**) Meandering index as a function of time for all cell lines, culture conditions, and phenotypes. The shaded area depicts the mean ± standard deviation range, and the center line within the shaded area represents the dynamics of the mean value. Table S1 contains the total numbers of colonies used for testing a difference in the mean value of meandering index between the groups of “good” and “bad” colonies; (**b**) ratio of the mean meandering index for “good” colonies to that for “bad” colonies.

We calculated the meandering index (*d*_net_/*d*_tot_) for each trajectory as a measure of colony motion persistence and investigated how it changed over time. This index takes values from 0 to 1. The minimum value corresponds to a complete absence of persistence, in which the colony returns exactly to the starting point. The maximum value corresponds to the complete persistence, in which the colony moves in one fixed direction. The dynamics of this index showed that the persistence decreased during the first 20–24 h and then stabilized at low levels (Figure 6a). Under the mTESR1/MG conditions, in contrast to the mobility measures described above, the phenotypes were discriminated uniformly across all cell lines in terms of the mean meandering index. “Good” colonies exhibited, on average, a higher motion persistence than the “bad” ones over time. At the same time, the lines demonstrated qualitatively different dynamics of the ratio of the mean meandering indices of “good” and “bad” colonies (Figure 6b). For CaSR colonies and mTESR1/MG conditions, this ratio had a local peak at approximately 12 h, at which the difference between phenotypes was most pronounced (*p* < 0.0001). After this time, the difference between phenotypes vanished for this line (*p* > 0.05 at 24 h). In contrast, the ratio for AD3 and H9 monotonously increased over a longer time range (Figure 6b), and AD3 colony phenotypes were distinguishable by the average meandering index over a longer time period (*p* < 0.01 at 12 h and 24 h; *p* < 0.0001 at 48 h; *p* < 0.001 at 55 h) than H9 colony phenotypes (*p* < 0.01 at 12 h and 24 h; *p* > 0.05 at 48 h and 55 h). There was no significant difference between phenotypes in terms of the mean meandering index under the E8/GT conditions (*p* > 0.05 for all times; Figure 6).

We obtained a similar picture of the relation between phenotypes in the analysis of the outreach ratio (*d*_max_/*d*_tot_), which characterized the compactness of the trajectories (Figure S6). This reflects the connection between the persistence of colony motion and the area covered during the motion.

#### 3.2.4. Velocity Autocorrelation Function

**Figure 7:**
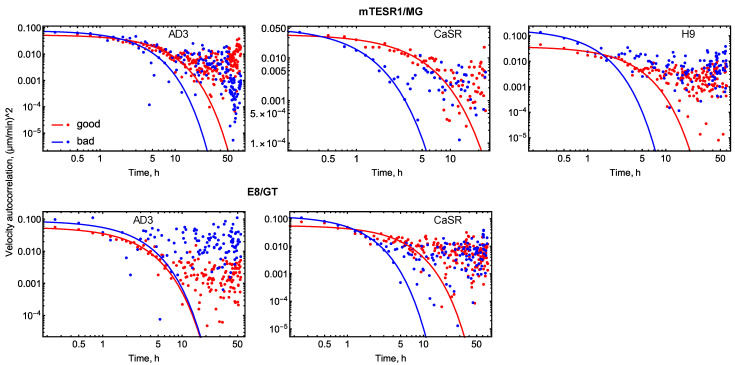
Velocity autocorrelation function in colony migration data (points) and an exponential approximation to the data (lines).

An important migration characteristic is the velocity autocorrelation function ρ(*t*), which shows the average correlation between the colony velocity vectors at time points separated by a time interval *t*. The velocity autocorrelation function calculated on our data exhibited different time dependence for different phenotypes in most cases (Figure 7). According to the model of normal diffusion, represented, for example, as the Ornstein–Uhlenbeck process, we should expect ρ(*t*) to exponentially decay with time. This means that correlation between velocities is only significant at short time intervals, reflecting a natural small time delay in changing velocity during the motion. The presence of such correlation at longer time intervals may indicate the action of additional mechanisms influencing the migration speed and direction and having a characteristic time scale comparable with the interval of high autocorrelation. An exponential approximation to our migration data showed good agreement with the data at early times but strongly deviated from them at later times (Figure 7). This suggests that hPSC colony migration may not follow normal diffusion, and alternative models are required to quantitatively characterize this migration.

### 3.3. Diffusion Models Characterizing hPSC Colony Migration

We applied two diffusion models to the migration data and used them to explain the dynamics of the mean squared displacement calculated from the data. The first model represented the normal diffusion scenario and was mathematically described as the classical Ornstein–Uhlenbeck stochastic process. According to this model, colony migration could be considered an ordinary Brownian motion. The second model represented possible anomalous properties of colony migration and was based on the fractional Klein–Kramers equation. This equation describes the dynamics of the probability of the colony to be at a given position at a given time and have a given velocity (see Section 2). The MSD functions theoretically derived from these models (Equations (1) and (2) in Section 2) predicted different time dependence of MSD. We fitted these functions to the MSD data to find a more relevant diffusion model and, thereby, to find evidence for either the presence or absence of anomalous diffusion properties in colony migration.

**Figure 8:**
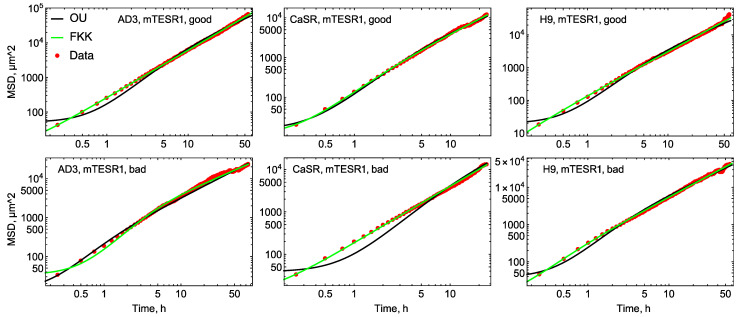
Dynamics of the mean squared displacement (MSD) in data (red dots) and in the best fits of two diffusion models, for the “good” (upper panels) and “bad” (bottom panels) phenotypes. OU, model based on the Ornstein–Uhlenbeck process (Equation (2) in Section 2); FKK, model based on the fractional Klein–Kramers equation (Equation (1) in Section 2). Results are shown for the mTESR1/MG culture conditions; results for E8/GT are shown in Figure S7.

We found that the FKK model described the MSD dynamics more accurately than the OU model, particularly at early times in most cases (Figure 8 and Figure S7). However, the MSD functions predicted by these models contained different numbers of free parameters that we used for fitting, with the FKK model having one more parameter. We used the Akaike information criterion (AIC) to decide which of the two models was more adequate in data approximation. This criterion provided a compromise between the quality of fit and the available number of degrees of freedom to achieve that quality. For the mTESR1/MG culture conditions, the FKK model performed better in terms of AIC for both “good” and “bad” colonies (Figure S8). The exception was the CaSR “good” colonies, for which the OU model exhibited a lower AIC value. We observed the reverse situation for the E8/GT conditions, for which the OU model outperformed the FKK model. Again, the CaSR “good” colonies were the only exception (Figure S8).

The MSD function from the FKK model has an asymptotic time dependence ~*t*^2–α^ at large times, where α is a parameter that defines the order of the fractional derivative in the model equation (see Section 2). As with other free parameters, the value of α is determined by fitting the MSD data. The MSD function from the OU model is a special case of that from the FKK model with α = 1 so that MSD in normal diffusion has the linear time dependence ~*t* at large times. Values of α ≠ 1 in the FKK model define the type of anomalous diffusion since MSD nonlinearly depends on time in the large time limit. A regime with α < 1 is called superdiffusive (MSD~*t^n^*, *n* > 1), so that MSD grows faster than in normal diffusion at large times. Values of α > 1 lead to MSD growing slower than in normal diffusion at large times (MSD~*t^n^*, *n* < 1), and this regime is called subdiffusive.

The parameter values obtained in the fits to the MSD data show that colonies whose migration is well explained by the FKK diffusion model exhibit mainly superdiffusive motion (Table 3). “Bad” AD3 colonies under mTESR1/MG and “good” CaSR colonies under E8/GT are the exceptions, which have α > 1 and, hence, show subdiffusive properties (Table 3 and Table 4). The difference in mobility measures between phenotypes described above is reflected, for example, in the variation of the mean thermal velocity *v*th of colonies. Under mTESR1/MG, “good” AD3 colonies have a larger value of this parameter than “bad” colonies, and the reverse is true for H9 colonies (Table 3). The direct comparison of other diffusion parameters between lines and phenotypes can be misleading since various values of α essentially influence the diffusive properties, particularly leading to different dimensionalities of parameters.

## 4. Discussion

The use of human pluripotent stem cells, including both human embryonic stem cells and induced pluripotent stem cells, in modeling various diseases and studying human development is currently widely discussed in the literature [22,23,24]. At the same time, the understanding of the biology and physiology of these cells is still incomplete.

Cellular movement as a complex process plays a critical role in diverse physiological and pathological processes, such as embryonic development, wound healing, immune responses, outgrowth, metastasis of cancer cells, and many others. At the same time, migration parameters and characteristics of hPSCs attract very little attention. This leads to the question of whether the migration properties of hPSCs can be attributed to their other important characteristics, like pluripotency and cell renewal. As an integral measure of numerous cellular and signaling processes involved in colony migration, one can expect that migration parameters are a valuable characteristic of the cells. However, opposite to the morphological parameters of hPSC colonies, which correlated well with the cell’s phenotype (“good” vs. “bad”) reflecting colony pluripotency status, migration characteristics for some lines discussed in the present study demonstrated less strait correlation with the colony morphology/phenotypes. This points to a need for a bigger cohort study. At the same time, taking into account the heterogeneous properties between various hPSC lines, this discovery can be further associated with different physiological characteristics and may determine some physical features of the lines.

Lack of knowledge hinders the development of reliable methods for maintaining hiPSCs in an undifferentiated state and the development of reliable differentiation protocols. In particular, this is true when methods are developed capable of operating with large volumes of cell cultures. The behavior of hiPSCs in culture is closely related to its morphological phenotype [7]. In recent years, the effort of many researchers, including our team, has been dedicated to developing machine-learning methods for recognizing the phenotypes of hPSC colonies in order to discriminate between “good” and “bad” clones in terms of their suitability to be maintained pluripotent and able to differentiate in many directions upon stimulation [4,5,6,13,25,26,27].

In the present study, we show that individual hiPSC colonies with “bad” morphology vs. “good” morphology have distinct migration parameters, and this is dependent upon cultivation conditions, which is in accordance with previously published data from Lin and colleagues [13]. We considered one of the first defined mediums, mTeSR1 [28], which contains protein components such as bovine serum albumin. Another culture medium used in our study was Essential 8 (E8), the Xeno-free, chemically defined media [29]. The choice of these culture media was based on the fact that both of them are the most widely used in research laboratories to maintain the pluripotent status of hPSCs. It is well established that human iPSC morphology depends on the cultivation conditions, such as medium; less defined media could induce cytoskeleton instability, while the cytoskeleton becomes more stable in the Xeno-free and defined media [30]. Harkness and colleagues revealed that the choice of media determines the lineage preference and, thus, the fate of the cell’s differentiation [30]. At the same time, the study of the migration characteristics of hPSCs under various cultivation conditions has attracted less attention than other aspects of hPSC physiology.

Generally, motility represents a convenient measure of the cellular function overall, as motility itself requires proper function of many cellular systems, such as the cytoskeleton, energy metabolism, plasma membrane plasticity, and various signaling cascades. However, on the basis of the present research, we cannot conclude that migration parameters possess the same strength for the estimation of the quality of hiPSC lines as the morphological phenotype. More research in this matter is needed to propose migration measurements as an essential criterion for quality control of hiPSCs for clinical application.

We focused on the analysis of migration characteristics of hPSC colonies with different morphological phenotypes by addressing parameters that reflect the colony speed and distance traveled (mobility measures), meandering index (motion persistence measures), outreach ratio (trajectory tortuosity characteristic), as well as the velocity autocorrelation function for two hiPSC lines (AD3 and CaSR) and hESC H9 line. Our analysis demonstrated variability of migration characteristics across hPSCs cultured in mTESR1/MG condition. At the same time, cells cultivated in the E8/GT condition showed a smoother behavior, i.e., we did not observe significant differences in migration between the two phenotypes. These results imply that, for mTESR1/MG, the migration characteristics of hPSC colonies can be used as a part of a general profile containing possible non-invasive markers of pluripotency status, while these characteristics are not informative for the Xeno-free E8 medium.

Our main discoveries suggest that colonies with “good” and “bad” morphological phenotypes exhibit different motility parameters, especially in the mTESR1/MG condition. It appears that migration speed does not differ among colonies with different phenotypes when they are grown in E8/GT, whereas the opposite was true when using mTESR1/MG. Additionally, under the E8/GT culture condition, no significant difference between phenotypes in terms of the mean meandering index was detected. In contrast to the mobility measures under the mTESR1/MG conditions, the phenotypes were discriminated uniformly across all cell lines in terms of the mean meandering index, with “good” colonies exhibiting a higher motion persistence than the “bad” ones over time. Therefore, the meandering index as a motion persistence measure complements the mobility measures and provides a distinctive variation pattern across cell lines.

It has previously been shown that cellular motion can be described in terms of the anomalous diffusion, demonstrating that it is important to account for the influence of biological processes involved in this process when characterizing migration [15]. Our results extend this approach as we consider colony migration, which can be viewed as emerging from the collective movement of many cells, and by taking into account different number of degrees of freedom in different diffusion models. The classical Ornstein–Uhlenbeck process [20] and the fractional Klein–Kramers equation [19] provide the models for the normal and anomalous diffusion, respectively. We showed that the colony mainly demonstrated the MSD dynamics consistent with the usual Brownian motion for the E8/GT conditions, while these dynamics were estimated as anomalous for most colonies under mTESR1/MG. The CaSR “good” colonies were the only exception from this result, which was probably related to the not-very-long dynamics for these colonies. Thus, we should be cautious when extending cell migration results to the colony level. Among the colonies with anomalous MSD dynamics under mTESR1/MG, the superdiffusive behavior was predominant, in accordance with previous findings about cellular migration [15].

Earlier it was suggested that the differences in hPSC migration might occur when some colonies start to deviate from the undifferentiated state, thus suggesting that the migration rate may represent a useful parameter to investigate spontaneous differentiation in culture [31,32]. Our group demonstrated that morphological parameters of cells and colonies of hPSCs represent a reliable measure of their pluripotency [4]. As such, colonies with the “bad” morphology may be regarded as deviations from pluripotency, being at the beginning of spontaneous differentiation. Herein, we have demonstrated that colonies with “bad” morphology, i.e., the colonies with lower level of pluripotency, exhibited migration behavior that is different from the “good” ones in the mTESR1/GT cultures.

Our ultimate goal is to combine morphological parameters with migration characteristics in our machine-learning models [4,5,6] for the selection of the best hPSC clone. The results presented here indicate that for this purpose it is better to employ mTERS1/MT cultures, since in E8/GT condition the differences in migration parameters between clones with “bad” and “good” phenotypes may be less obvious.

Our study has several limitations. We were able to analyze colony migration for only three cell lines and two culture conditions. Even though we could not establish a unique pattern of phenotypic difference in migration characteristics even for this constrained setup, more lines and conditions are desirable to make our conclusions more robust or to decipher more subtle patterns. Another limitation concerns different numbers of colonies involved in the analysis for different conditions, which is a consequence of differences in various factors associated with cell growth for different lines, phenotypes, and culture conditions. The small size of some samples weakens the conclusions about possible separation of “good” and “bad” colonies based on their migration characteristics. To equalize the colony statistics, more controlled experiments should be performed, which, on the other hand, demand more resources. The manual tracking of colony migration might be a source of possible undesirable artifacts in the processed colony trajectories and is time consuming. We plan to abandon it and develop a robust automated tracking method that would be efficient for the bright-field image analysis used in our study.

Despite the limitations, the present study demonstrates that measurement of the various parameters of the hPSC migration serves as a valuable tool providing information that may be useful for designing a model that may be used as a base for the automated control of the hPSC culture process.

## Figures and Tables

**Table 1:** Total number of trajectories under analysis for each cell line for two culture conditions (combinations of media/matrix) and two phenotypes. The data for H9 were used as an additional comparison with the data for the hiPSC lines and were collected only for mTESR1/MG.

Cell Line	Number of Trajectories			
	mTESR1/MG		E8/GT	
	“Good”	“Bad”	“Good”	“Bad”
AD3	1030	208	383	49
CaSR	213	60	84	108
H9	606	139	–	–

**Table 2:** Colony migration parameters. The colony trajectory is represented as a set of points (***r***_0_, ***r***_1_, …, ***r****_n_*, …, ***r****_N_*), where ***r****_i_* = (*x_i_*, *y_i_*) are coordinates of the colony center at *i*th time. The time interval ∆t between adjacent points on the trajectory equals 15 min. For simplicity of notation, we assume that at a given time *t* the colony resides at the point ***r****_n_*, so that t=n∆t. For more details, see Section 2.

**Parameter**	Definition	Description
Instantaneous colony velocity at time *t*	$v(t)=\frac{r_{n}-r_{n+1}}{∆t}$	Velocity vector ***v*** = (*v_x_*, *v_y_*) at time *t*
Mean colony speed over a time interval	$v=\langle \left|v(t)\right|\rangle$	Absolute value of the instantaneous velocity averaged over a given time interval
Net distance at time *t*	*d*_net_(*t*) = dist(***r***_0_, ***r****_n_*)	Distance between the initial point ***r***_0_ and the point ***r****_n_* at time *t*
Total distance up to time *t*	*d*_tot_(*t*) = $\sum_{i=0}^{n-1}dist(r_{i},r_{i+1})$	Sum of all distances covered by the colony, starting from the initial point ***r***_0_ and up to the point ***r****_n_* at time *t*
Maximum distance at time *t*	*d*_max_(*t*) = $\max_{0<\tau <t}d_{net}(\tau )$	Maximum distance from the initial point at which the colony center appears within the time interval (0, *t*)
Meandering index at time *t*	*d*_net_(*t*)/*d*_tot_(*t*)	This index represents the tortuosity of trajectories or persistence (also, linearity or straightness) of colony motion
Outreach ratio at time *t*	*d*_max_(*t*)/*d*_tot_(*t*)	This ratio characterizes the compactness of trajectory
Velocity autocorrelation function	$\rho \left(t\right)=\langle v(t+t_{0})\cdot v(t_{0})\rangle$	Scalar product of the instantaneous velocity vectors at time moments separated by delay *t*_0_, averaged over *t*_0_ and trajectories. This measure can be interpreted as the memory about the initial state that the migrating colony possesses at time *t*
Mean squared displacement at time *t*	$MSD\left(t\right)=\langle {\left|r_{n}-r_{0}\right|}^{2}\rangle$	Absolute value of the colony displacement from the initial state to the point ***r****_n_* at time *t*, squared and averaged over trajectories. This measure is used in the study of the type of diffusion exhibited by the colonies

**Table 3:** Parameter values from Equation (1) of Section 2 obtained by fitting to MSD data for the mTESR1/MG culture conditions. For CaSR “good” colonies, values from the OU model with the predefined α = 1 (Equation (2) of Section 2) are shown, since this model outperformed the FKK model in terms of AIC in this case (Figure S8). Means ± standard errors calculated from a set of independent fits are shown. The diffusion coefficient $D_{\alpha }$ is calculated from the fitted parameters: $D_{\alpha }=v_{th}^{2}/\gamma_{\alpha }$.

**Cell Line and Phenotype**	Parameter				
	α	$\gamma_{\alpha },1/h^{\alpha }$	$v_{th},\mu m/h$	σ, μm	$D_{\alpha },{\mu m}^{2}/h^{2-\alpha }$
AD3, “good”	0.652 ± 0.019	16 ± 13	34 ± 14	1.5 ± 0.7	86 ± 6
AD3, “bad”	1.168 ± 0.022	0.41 ± 0.08	7.9 ± 0.5	3.0 ± 0.1	153 ± 13
CaSR, “good”	1	0.51 ± 0.03	7.9 ± 0.2	1.9 ± 0.4	124 ± 1
CaSR, “bad”	0.690 ± 0.021	16 ± 13	29 ± 11	1.2 ± 0.8	60 ± 5
H9, “good”	0.689 ± 0.013	10 ± 2	21 ± 3	0.1 ± 0.3	46 ± 2
H9, “bad”	0.846 ± 0.005	5 ± 1	23 ± 2	1.2 ± 0.6	107 ± 2

**Table 4:** Parameter values from Equation (2) of Section 2 obtained by fitting to MSD data for the E8/GT culture conditions. For CaSR “good” colonies, values from the FKK model (Equation (1) of Section 2) are shown, since this model outperformed the OU model in terms of AIC in this case (Figure S8). Shown are means ± standard errors calculated from a set of independent fits; the standard error is 0.0 in most cases since the numerical optimization arrived at a unique minimum in all optimization runs. The diffusion coefficient $D_{\alpha }$ is calculated from the fitted parameters: $D_{\alpha }=v_{th}^{2}/\gamma_{\alpha }$.

**Cell Line and Phenotype**	Parameter				
	α	$\gamma_{\alpha },1/h^{\alpha }$	$v_{th},\mu m/h$	σ, μm	$D_{\alpha },{\mu m}^{2}/h^{2-\alpha }$
AD3, “good”	1	0.94 ± 0.00	11 ± 0.0	2.2 ± 0.0	133 ± 0.0
AD3, “bad”	1	0.94 ± 0.00	11 ± 0.0	4.1 ± 0.0	133 ± 0.0
CaSR, “good”	1.590 ± 0.004	0.12 ± 0.01	7.3 ± 0.2	1.9 ± 1.3	435 ± 9
CaSR, “bad”	1	2.94 ± 0.00	15 ± 0.0	1.1 ± 0.0	73 ± 0.0

## Data Availability

The data containing all trajectories extracted from the bright-field images were uploaded to the Zenodo public repository (https://zenodo.org/records/13936158, accessed on 25 October 2024); doi:10.5281/zenodo.13936158.

## Supplementary Materials

The following supporting information can be downloaded at https://www.mdpi.com/article/10.3390/life14111402/s1, supporting_information.pdf: A list of Figures S1–S8 and Table S1; Video S1: Motion of two migrating colonies (CaSR, mTESR1/MG culture conditions, 0–24 h) with the overlayed trajectories.
