# Supplementary material for: Human Pluripotent Stem Cell Colony Migration Is Related to Culture Environment and Morphological Phenotype
Source: Life (Basel). 2024 Oct 31;14(11):1402. doi: 10.3390/life14111402 (PMC11595361; doi:10.3390/life14111402)
Supplement: Supplementary file 1 [file life-14-01402-s001.zip › supporting_information_revised.pdf]

Supplementary Material

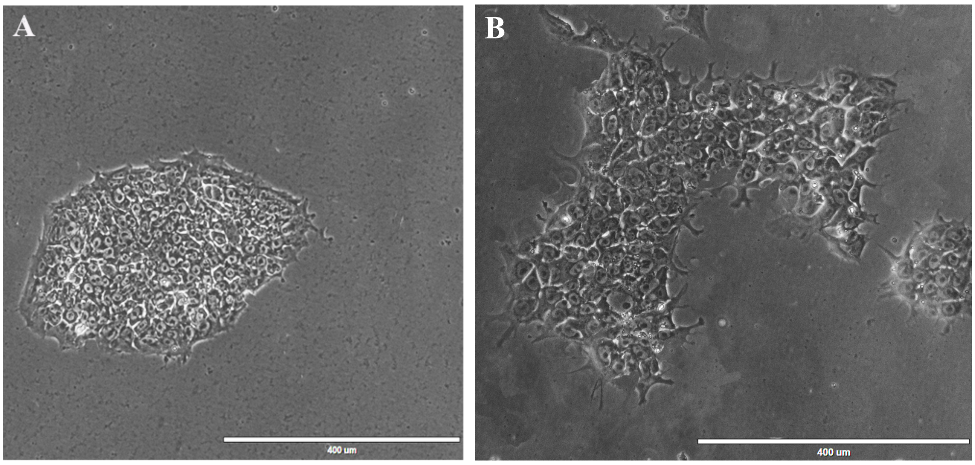

Figure S1. Examples of colonies with (A) “good” and (B) “bad” morphological phenotype (CaSR, mTESR1/MG culture conditions). All specific morphological differences between the two phenotypes are described in detail in Krasnova et al., International Journal of Molecular Sciences 2022, 23, 12902, doi:10.3390/ijms232112902.

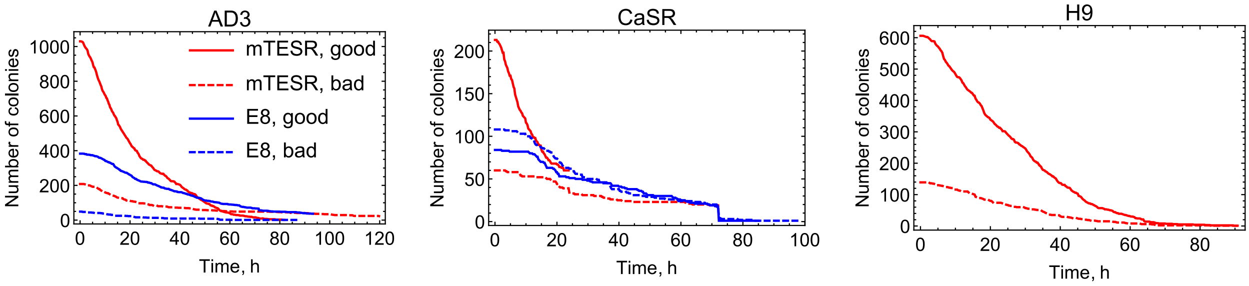

Figure S2. Total number of colonies under analysis as a function of time, for two media (mTESR and E8) and two phenotypes (good and bad). The number of tracked colonies reduced over time since the colonies were stopped to be tracked at the moment of merging with other colonies.

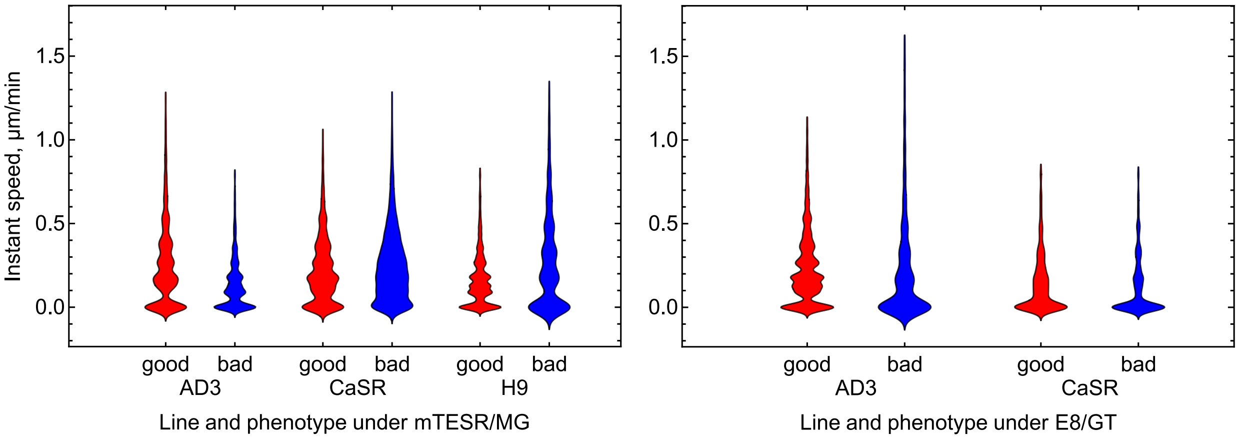

Figure S3. Violin plot showing distributions of instant speed values for colonies of three cell lines under two culture conditions.

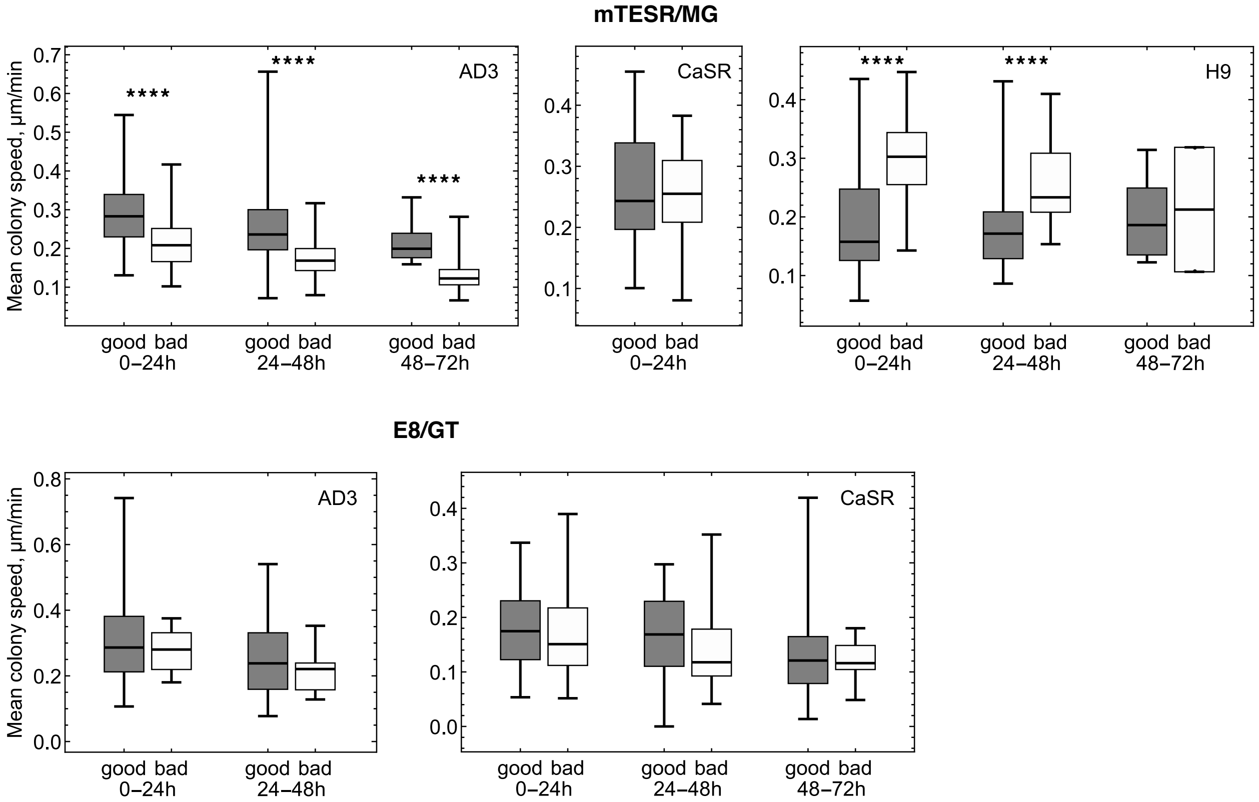

Figure S4. Box plots for the mean colony migration speed within three time intervals for all cell lines, culture conditions, and phenotypes. The averaging within a time interval was performed only over trajectories that lasted longer than the interval length. Statistically significant difference between phenotypes is marked by stars: $P < 0.0001$ (****). The absence of stars in the panels indicates statistically nonsignificant difference. The box plots for H9, mTESR/MG, 48–72h were plotted for only 7 good colonies and 2 bad colonies; no statistical tests were performed on these data.

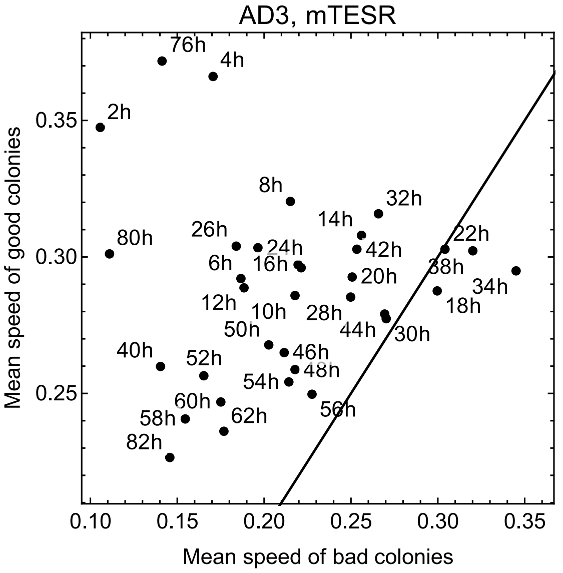

Figure S5. Scatter plot for the mean speed of good and bad colonies in the example of AD3 cell line and mTESR/MG growth conditions. Each point corresponds to a pair of good and bad colonies having exactly the same length, and this length is indicated next to the point. The solid line represents equality of the mean speeds.

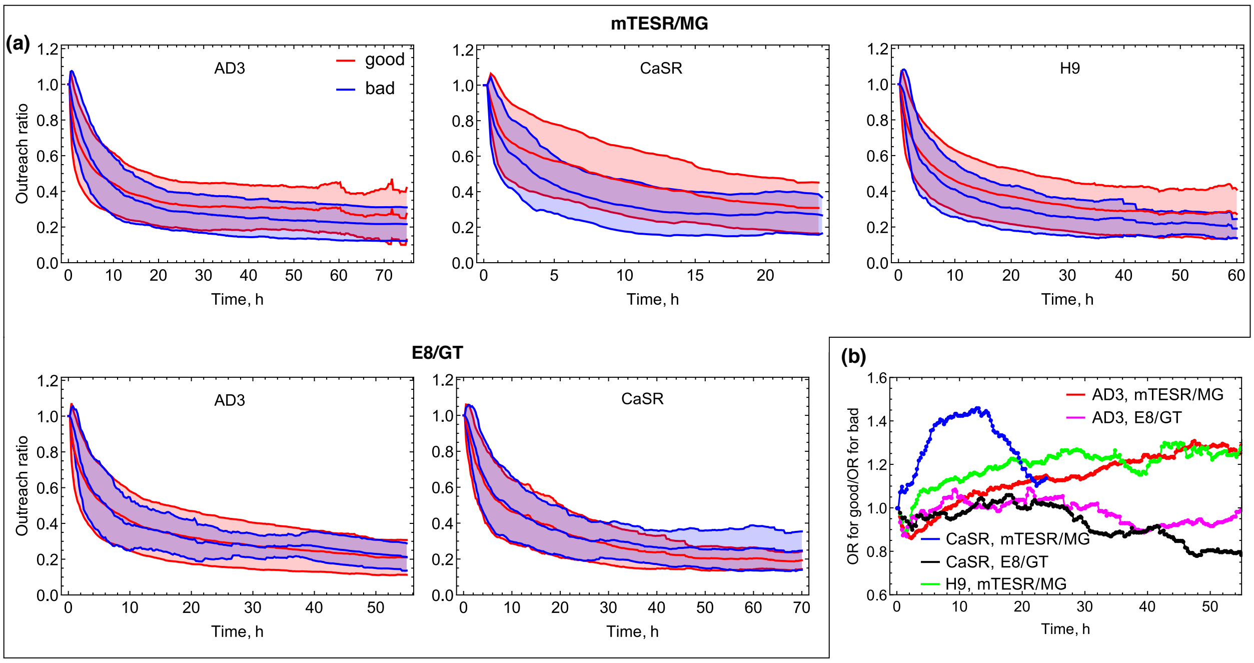

Figure S6. Dynamics of outreach ratio ($d_{\max}/d_{\mathrm{tot}}$) during migration. **(a)** Outreach ratio (mean ± standard deviation) as a function of time for all cell lines, culture conditions, and phenotypes; **(b)** Mean outreach ratio for good colonies divided by mean outreach ratio for bad colonies.

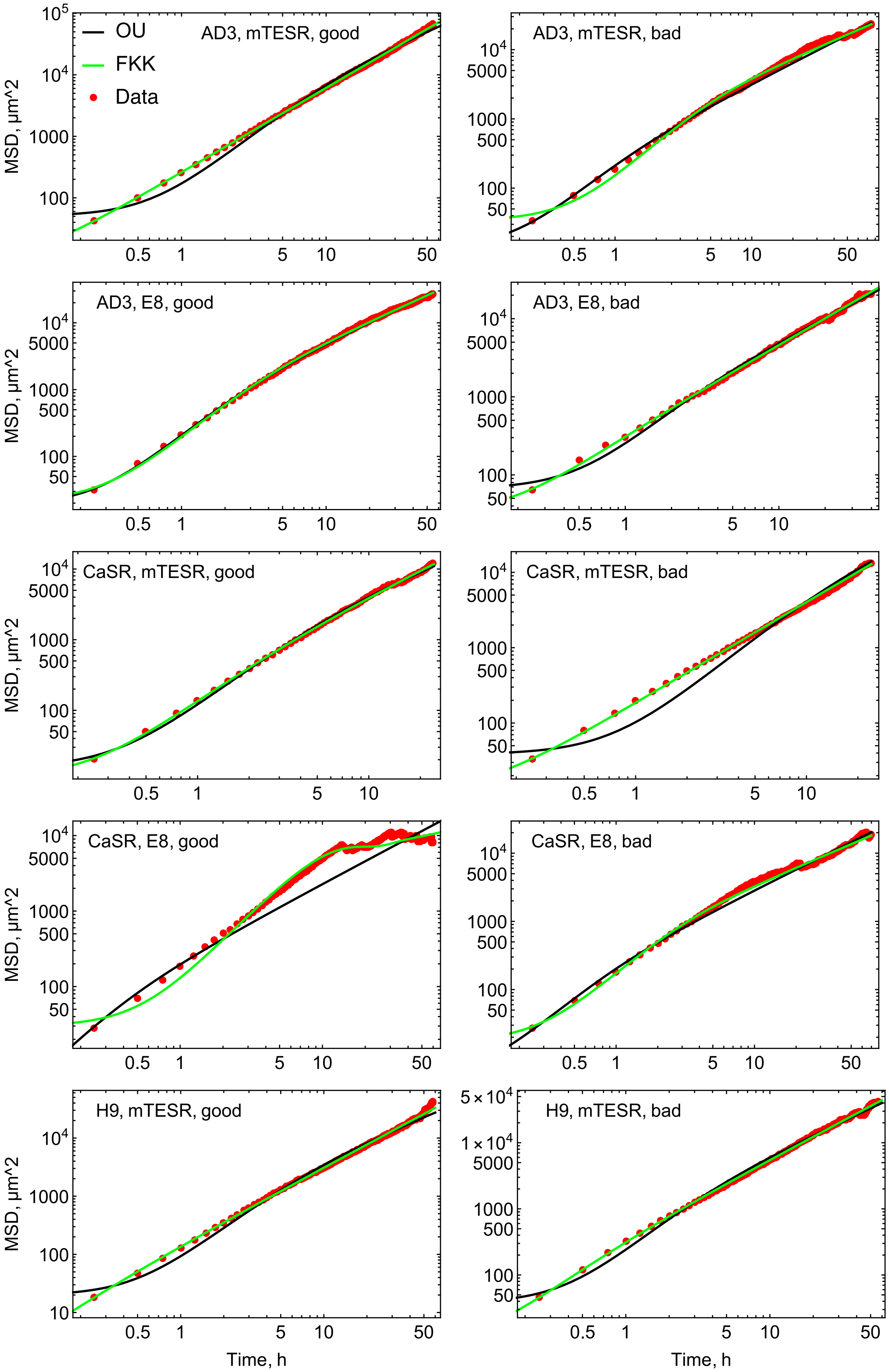

Figure S7. Dynamics of the mean squared displacement (MSD) in data (red dots) and in the best fits of two diffusion models, for the good (panels in the left) and bad (panels on the right) phenotypes. OU, model based on the Ornstein–Uhlenbeck process; FKK, model based on the fractional Klein–Kramers equation.

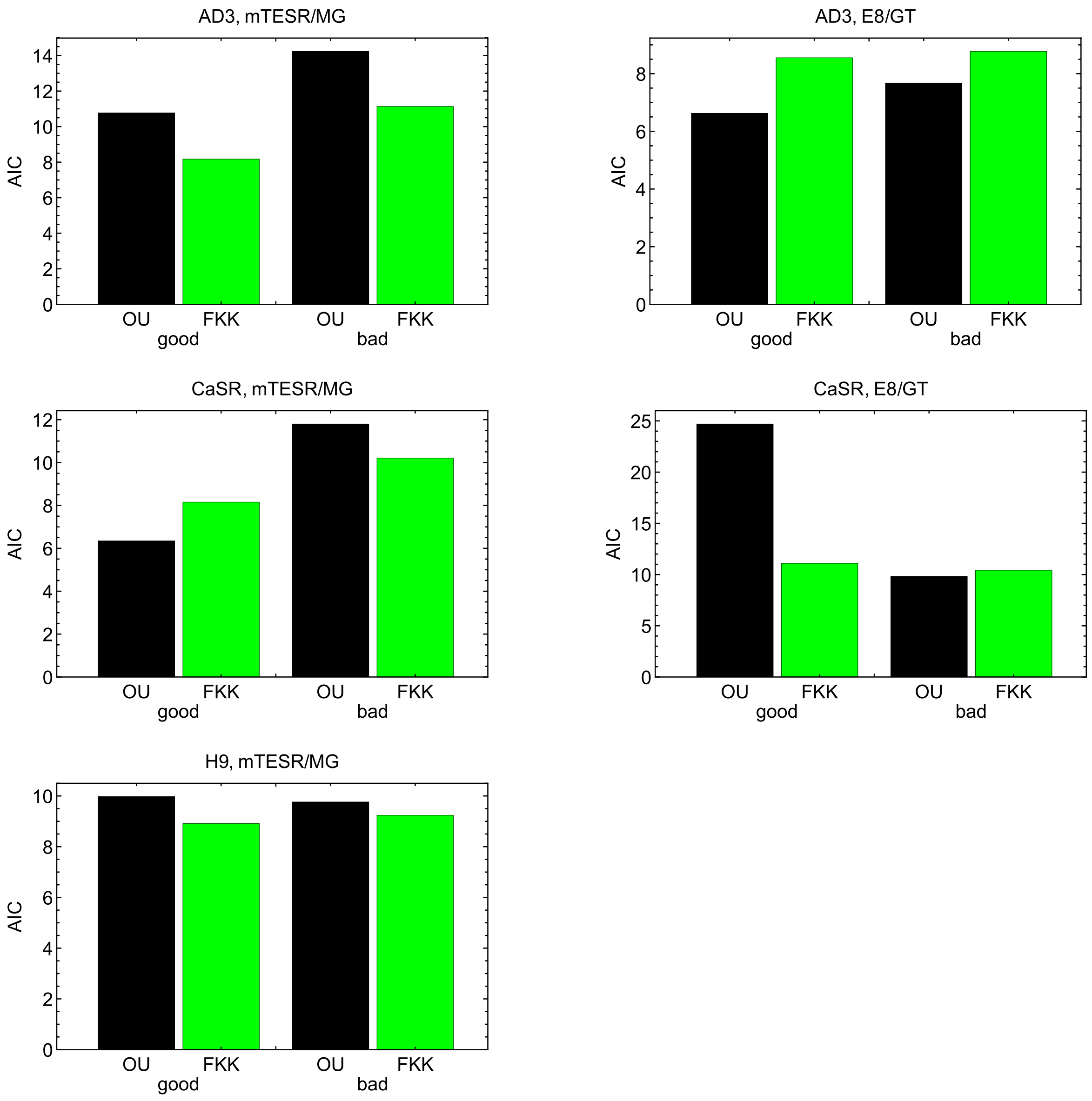

Figure S8. Akaike information criterion (AIC) values in the MSD data fitting by the MSD functions from the model based on the Ornstein–Uhlenbeck process (OU) and the model based on the fractional Klein–Kramers equation (FKK), for all cell lines, culture conditions, and colony phenotypes. Smaller AIC values correspond to better models.

Table S1. Total number of colonies used for testing a difference in the mean values of migration parameters in Figures 5 and 6 of the main text. The test was performed for the difference between the groups of "good" and "bad" colonies at four time points (12 h, 24 h, 48 h, and 55 h).

**Cell line**	**Number of colonies**							
	12 h		24 h		48 h		55 h	
	good	bad	good	bad	good	bad	good	bad
	mTESR1/MG							
AD3	664	150	369	97	123	59	70	55
CaSR	103	53	60	39	not used	not used	not used	not used
H9	464	111	302	70	75	19	49	23
	E8/GT							
AD3	331	32	227	16	128	9	102	7
CaSR	80	98	52	63	35	30	30	27
